# Changes in Traumatic Brain Injury Patterns Before and During the COVID-19 Pandemic: A Single-Center Study in Brazil

**DOI:** 10.7759/cureus.93758

**Published:** 2025-10-03

**Authors:** Matheus Guidini Lima, Francisco Pires, Leopoldo Mandic Ferreira Furtado, Mariana Gonçalves Lima De Paula, Rodrigo M Faleiro, Antonio Lucio Teixeira, Aline Silva De Miranda

**Affiliations:** 1 Neuroscience, Federal University of Minas Gerais, Belo Horizonte, BRA; 2 Surgery, Federal University of Minas Gerais, Belo Horizonte, BRA; 3 Pediatric Neurosurgery, Hospital Vila da Serra, Oncoclínicas, Nova Lima, BRA; 4 Medicine, Pontifícia Universidade Católica de Minas Gerais, Belo Horizonte, BRA; 5 Neurosurgery, Hospital João XXIII, Belo Horizonte, BRA; 6 Glenn Biggs Institute for Alzheimer’s and Neurodegenerative Diseases, University of Texas Health Science Center at San Antonio, San Antonio, USA; 7 Morphology, Federal University of Minas Gerais, Belo Horizonte, BRA

**Keywords:** alcohol, covid-19 pandemic, epidemiology, sport, traumatic brain injury

## Abstract

Traumatic brain injury (TBI) is a significant worldwide public health concern and is among the top causes of death in people under the age of 45 years. Research concerning the influence of the COVID-19 pandemic on TBI remains scarce, especially in underdeveloped nations. This retrospective observational study aimed to assess the impact of the COVID-19 pandemic on TBI in adults by comparing data from May 2019 (pre-pandemic) and May 2020 (pandemic period) at a tertiary trauma reference hospital in Brazil. The number of TBI cases was 458 in 2019 and 419 in 2020, with no statistically significant difference (p > 0.05). There was a decrease in sports-related TBIs (2.2% in 2019 vs. 0.5% in 2020, p-value = 0.03), but an increase in alcohol and/or drug use-related TBIs (18.3% in 2019 vs. 31.5% in 2020, p-value < 0.001). Statistical analyses were performed using Student’s t-test and the chi-square test. The pandemic altered the profile and incidence of TBI with respect to modifications of society’s habits that it has imposed. These results are useful when making policies for public health to deal with future global health emergencies. Study limitations include its retrospective design, which may introduce biases related to the data collection method and a lack of precision in medical record documentation. Additionally, the data analyzed refers only to the first month following the implementation of restriction measures; however, the sample size is considerable and provides meaningful insights into the initial impact of the pandemic.

## Introduction

The World Health Organization declared the COVID-19 pandemic on March 11, 2020 [1]. Isolation measures were introduced on a global scale, changing schools to online learning, remote work for companies, suppression of public and private events, closing businesses and non-essential activities, travel restrictions, and the mandatory use of face masks in public spaces. [2]. A national state of emergency was declared on March 13, 2020, in the United States. Similar to other countries, this led to the mobilization of hospital resources to address the growing demand for acute respiratory support, prompting various medical societies to issue recommendations for the redirection of resources [3].

Traumatic brain injury (TBI) refers to an injury caused by external mechanical forces to the head, leading to structural damage or functional impairment of the brain [4]. TBI is a significant contributor to morbidity and mortality among individuals with multiple injuries and ranks as a leading cause of death in those under 45 years old. Its causes include falls, motor vehicle accidents, sports-related injuries, interpersonal assaults, and others. TBIs are classified into two main types: closed (non-penetrating) and open (penetrating) [4].

TBI remains a major global public health concern due to its medical and socioeconomic impact. In the United States, approximately 214,000 hospitalizations were reported in 2020, and nearly 69,500 deaths occurred in 2021 [4,5]. Although TBI is also common in Brazil and has significant economic and social impacts, few epidemiological studies exist, making historical, geographical, and socioeconomic comparisons challenging [6]. One of the few epidemiological studies conducted in Brazil reported approximately 125,500 hospitalizations for TBI annually, around 9,715 in-hospital deaths, corresponding to a mortality rate of 5.1 per 100,000 inhabitants and a case fatality rate of 7.7% [7].

During the pandemic, many patients hesitate to seek emergency care for health concerns, increasing mortality and morbidity rates for acute medical conditions, such as heart attack and stroke [8]. It is uncertain whether these same trends were present for acute traumatic injuries. Neurological trauma is one of the leading causes of trauma-related hospitalizations, and one of the major challenges during the pandemic was balancing the need to conserve resources while preventing COVID-19 transmission and providing timely, high-quality care [9].

The objective of this study was to compare two similar periods, before and during the COVID-19 pandemic, assessing epidemiological variables related to TBI, with emphasis on incidence, severity, etiology, and associated factors. This study focuses on evaluating potential epidemiological changes in TBI during the COVID-19 pandemic. These observations can assist in directing public policies and healthcare resources to address future global crises, particularly in underdeveloped countries.

## Materials and methods

Study design

This is a cross-sectional and retrospective study conducted at Hospital João XXIII, located in the metropolitan region of Belo Horizonte. The study was approved by the Federal University of Minas Gerais and the hospital ethics committee (CAAE: 74492323.7.0000.5134).

Hospital João XXIII in Belo Horizonte, one of the largest emergency hospitals in Latin America, is the main trauma referral center in Minas Gerais, with approximately 400 beds and 13,000 monthly visits, and specializes in burns, polytrauma, and intoxications. Belo Horizonte, the state capital with over 20 million inhabitants, was recognized for its effective social isolation policies. On March 17, 2020, one day after confirming its first COVID-19 case, the city declared a public health emergency and established the COVID-19 Epidemic Response Committee. That same week, isolation measures were implemented, including the suspension of municipal school classes, the prohibition of public and private events with the potential for crowding, and, shortly thereafter, the closure of commerce and other nonessential activities.

Study setting and eligibility criteria

The sample was drawn from the database of patients screened at Hospital João XXIII between May 2019 and May 2020, representing the pre-pandemic and pandemic periods of COVID-19, respectively. During this period, 5,895 patients from all specialties were evaluated in May 2019, compared to 3,621 in May 2020. Data extraction was performed retrospectively through a detailed review of each electronic medical record from the specified periods. For this reason, in order to ensure a fair comparison, the same months before and during the pandemic were analyzed. May was chosen as it was marked by the most restrictive measures implemented during the pandemic in our study center, and it also corresponded to a period of ascending trends in COVID-19 cases and deaths in Brazil in 2020.

**Figure 1:**
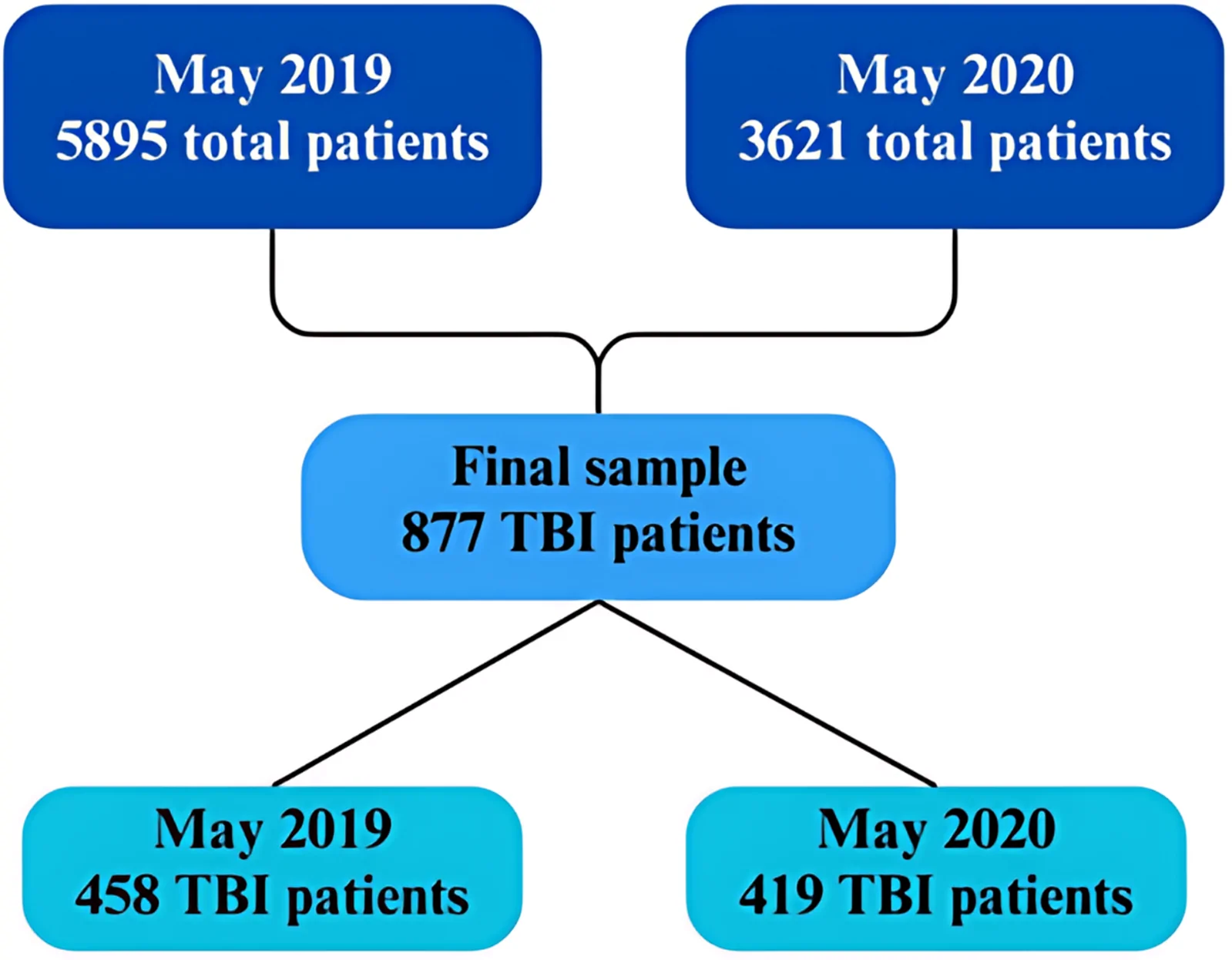
Flowchart of the study sample

The study included patients aged 18 years or older who were initially screened by the Neurology/Neurosurgery department or, in the case of polytrauma, by the trauma surgery team. Their medical records were subsequently reviewed, and after applying eligibility criteria, a final sample of 877 patients was obtained (Figure 1). Inclusion criteria were adults (≥18 years) treated at Hospital João XXIII in May 2019 or May 2020, with a documented TBI diagnosis and at least one initial trauma evaluation by a neurologist or neurosurgeon. Exclusion criteria comprised records lacking essential information on trauma mechanism, TBI severity, or clinical outcomes, as well as those with incorrect/inconsistent data or incomplete follow-up (e.g., patients discharged against medical advice or who did not complete treatment at Hospital João XXIII).

Data collection

Data related to the following variables were extracted from medical records: age, sex, race, ethnicity, city of origin, admission score on the Glasgow Coma Scale (GCS), trauma mechanism, preexisting comorbidities, Glasgow Outcome Scale (GOS) score [10], length of hospital stay, alcohol or drug use, reported abnormalities in cranial CT, use of antiplatelet or anticoagulant medications, and surgical approach, if performed. Because surgical indications were beyond the scope of this study, they were not addressed in detail. Such indications are inherently broad and highly specific, relying on multiple clinical and imaging criteria.

When information regarding comorbidities or medication use was not available (either due to the patient’s lack of knowledge or the impossibility of collecting such information), the term “not informed” was recorded, and these data were employed in the statistical analysis. Alcohol or drug use was documented at patient admission when actively inquired about by any of the physicians providing care and reported in the medical records, either through patient self-report or clinical assessment of signs of intoxication.

Trauma severity was defined according to GCS scores: 13-15 as mild, 9-12 as moderate, and 3-8 as severe. Outcomes were assessed using the GOS, with categories defined as follows: 1 - death; 2 and 3 (grouped) - severe disability; 4 - moderate disability; and 5 - mild disability.

Statistical analysis

Statistical analysis was conducted using IBM SPSS Statistics for Windows, Version 26.0 (Released 2018; IBM Corp., Armonk, NY, USA) and Excel Office 2013 (Microsoft Corporation, Redmond, WA, USA). Data were presented descriptively, with absolute and relative frequencies used for qualitative variables. Parametric statistical tests were applied as the normality of the quantitative outcome variables was assessed using the Shapiro-Wilk test (N ≥ 100), which indicated that the data followed a normal distribution. Parametric tests are generally more powerful in detecting significance.

The 2019 and 2020 groups were compared for all quantitative variables using the mean and the Student’s t-test. The chi-square test was used to analyze the distribution of qualitative variables based on their relative frequency. A significance level of 0.05 (5%) was set for this study.

Regarding surgical procedures, percentages were calculated based on the total number of individuals who underwent each type of surgery per group. It is important to note that each patient could undergo more than one type of procedure during the same surgical session, such as draining an acute subdural hematoma (ASDH), performing a decompressive craniectomy (DC), and implanting an intracranial pressure monitoring device. Therefore, the sum of percentages exceeds 100%.

## Results

After applying the inclusion and exclusion criteria, 877 patients were selected from May 1 to 31, 2019, and May 1 to 31, 2020. A power analysis performed with GPower 3.1.9.4 (α = 0.05) indicated a power of 0.902 for the sample, exceeding the commonly accepted threshold of 0.80, with a significance level of 0.05.

A non-statistically significant reduction (8.5%) in the number of TBI cases was observed during the pandemic (458 cases in 2019 vs. 419 in 2020). The groups also had similar age, admission GCS score, and length of hospital stay. As shown in Table 1, a higher prevalence of TBI was observed in the younger population in both groups.

**Table 1:** Distribution and comparison of quantitative factors between groups The Student’s t-test was used.

Statistic	Age		Glasgow at admission		Length of hospital stay	
	May 2019	May 2020	May 2019	May 2020	May 2019	May 2020
Mean	47.34	48.66	14.36	14.33	5.4	5.64
Median	43	46	15	15	2	2
SD	20.34	19.91	1.91	1.41	13.71	13.06
CV	43%	41%	13%	10%	254%	232%
Min	18	18	3	3	1	1
Max	105	94	15	15	138	170
N	458	419	458	419	458	419
CI	1.86	1.91	0.18	0.14	1.26	1.25
p-Value	0.33		0.83		0.79	

The qualitative variables are listed in Table 2, where some associations with statistically significant p-values were found. Regarding the need for cranial CT scans upon admission, 9% of TBIs in 2019 did not undergo the procedure compared to 4.1% in 2020 (p = 0.004, RR = 0.60, CI = 0.42-0.85). More patients in the 2019 group were discharged without neuroimaging studies. Despite this difference, the samples were similar regarding secondary tomographic findings related to TBI, including intracranial hematomas or skull fractures (18.3% in 2019 vs. 19.1% in 2020). No significant differences were found for variables like mortality, TBI severity, length of hospital stay, and surgical intervention submission (Table 1).

**Table 2:** Distribution and comparison of the frequency of qualitative factors between groups ^*^ Statistically significant (p < 0.05) The chi-square test was used. TBI: traumatic brain injury

Variable		May 2019 (before pandemic), n (%)	May 2020 (during pandemic), n (%)	Total, n (%)	Relative risk (95% CI)	p-Value
Sex	Female	166 (36.2)	133 (31.7)	299 (34.1)		0.16
	Male	292 (63.8)	286 (68.3)	578 (65.9)	1.11 (0.96, 1.29)	
TBI severity	Mild	431 (94.1)	395 (94.3)	826 (94.2)		0.92
	Moderate	14 (3.1)	18 (4.3)	32 (3.6)	1.18 (0.84, 1.65)	0.33
	Severe	13 (2.8)	6 (1.4)	19 (2.2)	0.66 (0.37, 1.18)	0.15
Previous comorbidities	No	213 (46.5)	200 (47.7)	413 (47.1)		0.72
	Not informed	78 (17.0)	47 (11.2)	125 (14.3)	0.78 (0.62, 0.98)^*^	0.01^*^
	Yes	167 (36.5)	172 (41.1)	339 (38.7)	1.05 (0.91, 1.21)	0.16
Length of hospital stay	Up to 24 hours	198 (43.2)	171 (40.8)	369 (42.1)		0.47
	24-48 hours	122 (26.6)	110 (26.3)	232 (26.5)	1.02 (0.86, 1.22)	0.90
	More than 48 hours	138 (30.1)	138 (32.9)	276 (31.5)	1.08 (0.92, 1.27)	0.37
Outcome	Death	20 (4.4)	11 (2.6)	31 (3.5)	0.74 (0.48, 1.13)	0.16
	Non-death	438 (95.6)	408 (97.4)	846 (96.5)		
Trauma mechanism	Animal accident	2 (0.4)	1 (0.2)	3 (0.3)	0.66 (0.17, 2.61)	0.62
	Motor vehicle accident	162 (35.4)	132 (31.5)	294 (33.5)	0.89 (0.75, 1.05)	0.23
	Assault	46 (10.0)	54 (12.9)	100 (11.4)	1.07 (0.86, 1.33)	0.19
	Fixed object collision	3 (0.7)	0 (0.0)	3 (0.3)	-	0.09
	Sharp and/or firearm injury	2 (0.4)	3 (0.7)	5 (0.6)	1.19 (0.53, 2.65)	0.58
	Unknown	0 (0.0)	1 (0.2)	1 (0.1)	1.98 (0.51, 7.67)	0.30
	Fall from standing height	139 (30.3)	142 (33.9)	281 (32.0)		0.26
	Fall from heights	83 (18.1)	79 (18.9)	162 (18.5)	0.97 (0.79, 1.17)	0.78
	Object falling on the head	11 (2.4)	5 (1.2)	16 (1.8)	0.62 (0.33, 1.16)	0.18
	Sports-related injuries	10 (2.2)	2 (0.5)	12 (1.4)	0.33 (0.13, 0.85)^*^	0.03^*^
Submitted to the surgical procedure	No	442 (96.5)	404 (96.4)	846 (96.5)		0.95
	Yes	16 (3.5)	15 (3.6)	31 (3.5)	1.01 (0.70, 1.47)	
Brain CT scan alteration	No	333 (72.7)	322 (76.8)	655 (74.7)		0.16
	Did not perform	41 (9.0)	17 (4.1)	58 (6.6)	0.60 (0.42, 0.85)^*^	<0.01^*^
	Yes	84 (18.3)	80 (19.1)	164 (18.7)	0.99 (0.83, 1.18)	0.76
Use of alcohol or drugs	No	374 (81.7)	287 (68.5)	661 (75.4)		<0.01^*^
	Yes	84 (18.3)	132 (31.5)	216 (24.6)	1.41 (1.21, 1.63)^*^	
Use of antiplatelet or anticoagulant	No	390 (85.2)	351 (83.8)	741 (84.5)		0.57
	Not reported	51 (11.1)	44 (10.5)	95 (10.8)	0.98 (0.78, 1.23)	0.76
	Yes	17 (3.7)	24 (5.7)	41 (4.7)	1.24 (0.92, 1.66)	0.16

Concerning trauma mechanisms, there was a significant reduction in sport-related TBI (2.2% in 2019 vs. 0.5% in 2020; p = 0.03, RR = 0.33, CI = 0.13, 0.85). No statistical differences were found for the other trauma mechanisms between the two groups. Another noteworthy finding was the higher use of alcohol and/or drugs in the context of TBI during the pandemic (31.5% in 2020 versus 18.3% in 2019; p < 0.01, RR = 1.41, CI = 1.21, 1.63). It is important to emphasize that all statistically significant results had confidence intervals not crossing 1, indicating significant associations with good estimate precision.

In 2019, 20 (4.4%) of patients died during hospital stay, compared to a lower rate of 11 (2.6%) in 2020. However, this difference was not statistically significant. When categorizing patients by outcome types, as classified by the GOS (Table 3), and severity of TBI, there were no differences between study groups.

**Table 3:** Distribution and comparison of functional outcomes (using the GOS scale: 1 - death; 2 and 3 (grouped) - severe disability; 4 - moderate disability; and 5 - mild disability), with TBI categorization, between groups The chi-square test was used. GOS: Glasgow Outcome Scale; TBI: traumatic brain injury

TBI severity		May 2019 (before pandemic), n (%)	May 2020 (during pandemic), n (%)	Total	Relative risk (95% CI)	p-Value
Severe TBI	Death	8 (61.5)	3 (50.0)	11 (57.9)	0.41 (0.10, 1.64)	0.64
	Severe disability	2 (15.4)	1 (16.7)	3 (15.8)	0.50 (0.09, 2.64)	0.94
	Moderate disability	2 (15.4)	0 (0.0)	2 (10.5)	-	0.31
	Mild disability	1 (7.7)	2 (33.3)	3 (15.8)	-	0.15
Moderate TBI	Death	5 (35.7)	3 (16.7)	8 (25.0)	0.56 (0.24, 1.30)	0.22
	Severe disability	0 (0.0)	2 (11.1)	2 (6.3)	1.50 (0.66, 3.40)	0.20
	Moderate disability	4 (28.6)	3 (16.7)	7 (21.9)	0.64 (0.28, 1.46)	0.42
	Mild disability	5 (35.7)	10 (55.6)	15 (46.9)		0.27
Mild TBI	Death	7 (1.6)	5 (1.3)	12 (1.5)	0.87 (0.46, 1.65)	0.67
	Severe disability	3 (0.7)	1 (0.3)	4 (0.5)	0.52 (0.13, 2.11)	0.36
	Moderate disability	4 (0.9)	7 (1.8)	11 (1.3)	1.33 (0.78, 2.28)	0.29
	Mild disability	417 (96.8)	382 (96.7)	799 (96.7)		0.97
Total	Death	20 (4.4)	11 (2.6)	31 (3.5)	0.74 (0.48, 1.13)	0.16
	Severe disability	5 (1.1)	4 (1.0)	9 (1.0)	0.92 (0.45, 1.87)	0.84
	Moderate disability	10 (2.2)	10 (2.4)	20 (2.3)	1.04 (0.66, 1.63)	0.84
	Mild disability	423 (92.4)	394 (94.0)	817 (93.2)		0.33

Regarding surgeries for conditions secondary to TBI (Table 4), ICH drainages were more frequent in 2020 (33.3% vs. 6.3%), with fewer DCs and ASDH drainages; however, no statistically significant differences were observed (p = 0.06).

**Table 4:** Distribution and comparison of surgeries performed between groups The chi-square test was used. AEDH: acute epidural hematoma; ASDH: acute subdural hematoma; CSDH: chronic subdural hematoma; DC: decompressive craniectomy; EVD: external ventricular drainage; ICPM: intracranial pressure monitoring; IPH: intraparenchymal hematoma

Procedure	May 2019 (before pandemic), n (%), N = 16	May 2020 (during pandemic), n (%), N = 15	p-Value
Skull depression correction	2 (12.5)	2 (13.3)	0.96
DC	4 (25.0)	1 (6.7)	0.17
IPH drainage	1 (6.3)	5 (33.3)	0.06
EVD	0 (0.0)	1 (6.7)	0.29
AEDH drainage	4 (25.0)	3 (20.0)	0.74
ASDH drainage	6 (37.5)	2 (13.3)	0.12
CSDH drainage	2 (12.5)	1 (6.7)	0.58
ICPM implant	5 (31.3)	3 (20.0)	0.47
Treatment of CSF fistula	1 (6.3)	0 (0.0)	0.33

## Discussion

The impact of the COVID-19 pandemic on trauma care and epidemiology has been reported across different healthcare contexts, but findings remain heterogeneous. For instance, a study from New Zealand compared two 14-day periods before and during the national lockdown and found a reduction in both the number and severity of trauma admissions [11]. Similarly, a Canadian study observed a decline in pediatric emergency room visits for injuries, particularly those related to vehicle accidents and sports activities during the lockdown [12]. A Peruvian study at a tertiary trauma center also revealed a marked reduction in hospital admissions during the first two months of the pandemic. Compared to March and April 2019, the number of patients treated decreased by 55.8% and 88.6%, respectively, although some categories of traumatic conditions remained either stable or decreased at varying rates [13].

Only one Brazilian study compared the epidemiology of trauma-related conditions before and during the pandemic. Ferreira Furtado et al. analyzed pediatric TBI and found an overall reduction in ER admissions. However, recreational causes of pediatric TBI increased during the first year of the pandemic, with a significant rise in bicycle-related falls [14].

The COVID-19 pandemic caused an unprecedented global health crisis, with consequences that extend beyond the morbidity and mortality related to the virus itself. There were also several behavioral changes at the population level, including increased alcohol and drug consumption, as observed in the present study, which showed a significant increase in alcohol- or drug-related TBI cases. In contrast, there was a reduction in sport-related TBI. These results could be relevant for future pandemics and the management of the mental health of the population within this context [15].

Alcohol consumption has long been recognized as a risk factor for TBI, with a robust association between alcohol consumption and TBI cases in emergency departments [16]. It is also important to note that there are several mechanisms by which alcohol can exacerbate secondary brain damage. Alcohol can reduce cerebral blood flow, and the byproducts generated by alcohol metabolism can impair the stability of capillary membranes, potentially intensifying the degree of brain injury caused by TBI [17].

Previous studies on global crises (e.g., terrorist events such as 9/11, epidemic outbreaks such as SARS in 2003, and economic crises such as the 2008 Great Recession) have indicated that there were no significant changes in alcohol use among the general population. This apparent stability resulted from a decrease in alcohol consumption among certain groups, while more vulnerable subgroups exhibited an increase, including men, young single individuals, and those with lower educational attainment or with depressive or anxiety disorders [18]. During the COVID-19 pandemic, the literature revealed mixed trends in alcohol consumption. In Brazil, Malta et al. conducted a survey of 45,161 individuals, reporting a 17.6% increase in alcohol consumption among adults during social restrictions, with the highest increase among those aged 30-39, an age range associated with increased risk for TBI [19].

Regarding illicit drug use, its impact also depends on the demographic context and the specific substance. Increased drug use during the pandemic was associated with a higher risk of severe COVID-19 infection or hospitalization [20]. There was a rise in stimulant (e.g., cocaine and methamphetamine) overdose deaths in 2020 [21]. Additionally, marijuana use has increased in several countries, including the US (43.9%) [22]. In Brazil, Nin et al. reported that individuals who perceived themselves as practicing lower levels of social distancing increased their drug use, while those with higher levels of social distancing significantly reduced it. Anxiety, depression, and sociodemographic factors (e.g., being male, low income, and low education) were associated with higher patterns of drug use [23]. Increased substance-related injuries across a population may result from a confluence of environmental changes/triggers, individual behavioral choices, and societal factors, as postulated by public health and behavioral epidemiology models [24]. TBI and substance use display a bidirectional relationship. TBI is associated with impaired executive function and impulsivity, which increases risk-taking behavior and substance abuse. Conversely, individuals with histories of substance use are at greater risk for sustaining TBI [25].

An American study highlighted differences in TBI patterns, showing that falls were more frequent before the pandemic (61.4% vs. 40.8%), while traffic accidents (5% vs. 18.4%) and interpersonal assaults (9.9% vs. 12.2%) were more common during the pandemic [26]. In our analysis, trauma mechanisms were similar between groups, except for sports-related TBIs, which significantly decreased during the pandemic. The exact annual incidence of sports-related TBI is unknown, but it is estimated to range from 300,000 to 3.8 million cases annually in the United States [27]. This reduction probably reflects the impact of social isolation on leisure activities. Similarly, a multicenter US study reported a decline in recreational trauma cases in general from 3% to 1.7% during the pandemic [28].

The number of patients discharged without undergoing a head CT scan decreased, resulting in a higher proportion of CT scans performed during the pandemic on patients with TBI. A number of studies showed an upward trend in the performance of cranial and chest CT scans in the emergency setting during the pandemic. In 2020, despite a decline in the overall number of patients admitted to emergency departments, CT utilization increased more than twelvefold compared to 2019 [29]. Several factors have been associated with the overuse of imaging studies, including lack of physician experience, pressure to reduce patient waiting times, excessive workload that hinders the development of a trusting patient-provider relationship, patient anxiety about possible results, fear of legal action, and the influence of colleagues on duty, among others. These issues were compounded by the uncertainty surrounding the public health crisis at the beginning of the pandemic, which likely led more physicians to request imaging examinations without a clear clinical indication.

This study has limitations. Being retrospective, it may introduce biases due to the data collection method and the lack of precision in documenting patient characteristics. For instance, patients may omit information, or the evaluating physician may fail to record details in the medical records. Alcohol or drug use, in particular, is often sensitive to these variations. Relevant variables, including ICU admission, emergency response time, and time to hospital presentation, were not reported. Direct behavioral data were also not collected. Moreover, the data analyzed refer to only a single month following the implementation of population restriction measures during the pandemic, which represents a temporal limitation, as other trend changes may have occurred during subsequent waves of infection. Finally, as the data derive from a single tertiary center in Brazil, the generalizability of the findings is limited, particularly across different geographic or socioeconomic contexts. Multicenter studies, including urban-rural comparisons, are warranted to better capture broader epidemiological trends.

Despite these limitations, the present study is supported by a robust and large sample size. The data set allows for a comprehensive analysis of TBI presentation and outcomes, which strengthens the reliability of the findings. Moreover, the results open the gate to more focused local and regional studies. The pandemic varied according to region, and some vulnerable groups, including the elderly, women, and essential workers (e.g., healthcare and public safety professionals), were more likely to be exposed to certain risk scenarios [30]. Future studies could focus on specific populations to develop more tailored approaches. The information gathered in this study during the pandemic could serve as an incentive for similar research, even beyond the pandemic context.

## Conclusions

This article is the first in the Latin American literature to describe the factors associated with epidemiological changes in the profile of TBI during the pandemic. The alarming data on alcohol and drug use among vulnerable populations during pandemics, and also during major economic recessions marked by rising unemployment rates, deserve special attention. As shown, the cases of TBI related to this issue increased considerably. It is worth mentioning that in Brazil, no restrictions were imposed on alcohol/drug availability during the pandemic. Treatment facilities and pathways for alcohol and drug involvement should be maintained at these times, as well as regular prevention to reduce not only TBI effects but also other comorbidities.
